# Shaping of Looped Miniaturized Chalcogenide Fiber Sensing Heads for Mid-Infrared Sensing

**DOI:** 10.3390/s141017905

**Published:** 2014-09-26

**Authors:** Patrick Houizot, Marie-Laure Anne, Catherine Boussard-Plédel, Olivier Loréal, Hugues Tariel, Jacques Lucas, Bruno Bureau

**Affiliations:** 1 Verres et Céramiques, ISCR UMR-CNRS 6226, Université de Rennes 1, Rennes 35042, France; E-Mails: patrick.houizot@univ-rennes1.fr (P.H.); mlanne22@hotmail.fr (M-L.A); catherine.boussard@univ-rennes1.fr (C.B.-P.); jacques.lucas@univ-rennes1.fr (J.L.); 2 Mécanique et Verres, IPR UMR-CNRS 6251, Université de Rennes 1, Rennes 35042, France; 3 INSERM UMR991, Université de Rennes 1, Rennes 35033, France; E-Mail: olivier.loreal@univ-rennes1.fr; 4 DIAFIR, Le Gallium, 80 Avenue des Buttes de Coesmes, Rennes 35700, France; E-Mail: hugues.tariel@diafir.com

**Keywords:** chalcogenide glass, optical fiber, mid-infrared spectroscopy, sensing head shaping, miniaturization, endoscopy

## Abstract

Chalcogenide glass fibers are promising photonic tools to develop Fiber Evanescent Wave Spectroscopy (FEWS) optical sensors working in the mid-infrared region. Numerous pioneering works have already been carried out showing their efficiency, especially for bio-medical applications. Nevertheless, this technology remains confined to academic studies at the laboratory scale because chalcogenide glass fibers are difficult to shape to produce reliable, sensitive and compact sensors. In this paper, a new method for designing and fabricating a compact and robust sensing head with a selenide glass fiber is described. Compact looped sensing heads with diameter equal to 2 mm were thus shaped. This represents an outstanding achievement considering the brittleness of such uncoated fibers. FEWS experiments were implemented using alcoholic solutions as target samples showing that the sensitivity is higher than with the routinely used classical fiber. It is also shown that the best compromise in term of sensitivity is to fabricate a sensing head including two full loops. From a mechanical point of view, the breaking loads of the loop shaped head are also much higher than with classical fiber. Finally, this achievement paves the way for the use of mid-infrared technology during *in situ* and even *in vivo* medical operations. Indeed, is is now possible to slide a chalcogenide glass fiber in the operating channel of a standard 2.8 mm diameter catheter.

## 1. Introduction

Chalcogenide glasses are original materials built up using the chalcogens, *i.e.*, sulfur, selenium or/and tellurium, mixed with other elements close to them in the periodic chart such as arsenic, antimony, germanium, *etc.* The main motivation to develop these vitreous species concerns their remarkably wide transmission window [1,2], which extends in the infrared range from 2 to 20 μm, depending on the glass compositions, whereas classical silica glasses stop transmitting light at 2 µm. Moreover, due to their vitreous state, these glasses are quite easy to shape into optical devices by thermoforming. Thus, chalcogenide glasses are matchless materials for the development of optical sensors working in the mid-infrared region [3,4]. At this time, the most effective application at an industrial level concerns the development of molded lenses for thermal imaging systems. Indeed, they have been developed as an alternative to lenses built with germanium crystals which are much more expensive and difficult to fabricate. Another promising application concerns the drawing of chalcogenide glasses into optical fibers in order to detect, monitor and characterize organic (bio-) molecules [3–7]. Indeed, optical sensors operating in the mid-IR region, where the main IR signatures of molecules and biomolecules are located, are playing an important role in the development of analytical techniques giving *in situ* information on metabolism mechanisms [8–11]. Fiber Evanescent Wave Spectroscopy, called FEWS, is an efficient and easy to implement way to collect these infrared signatures. Moreover, FEWS enables *in situ* and in real time studies with no sampling. Numerous exploratory works have been carried out in different application domains, such as: detection of pollutants in waste water [12,13], following chemical or industrial processes [14–16], detection of bacterial contaminations in food [17], monitoring of bacterial biofilm spread [18], study of tumorous tissues [19,20], and of biological liquids such as serum, blood or plasma [21]. These promising results led to the foundation of a company, DIAFIR (Rennes, France), working on the shaping of chalcogenide glass fiber for bio-medical applications.

The main difficulties encountered in the use of these optical fibers reside in their relative fragility on the one hand, and in the lack of compactness of the measuring head on the other hand. To improve the sensitivity together with the compactness of the sensing head, some tapered fibers were realized, but in any case the bending radius of this fiber in service remains high, around 15 mm for a 100 µm fiber diameter [22,23]. In this paper, a new method for designing and fabricating compact and robust sensing heads is described. The FEWS measurement sensitivity of the new chalcogenide glass fiber heads was evaluated, together with their mechanical behavior.

## 2. Experimental Section

### 2.1. Fiber Evanescent Wave Experimental Set-Up

Previous application works, carried out in a multidisciplinary frame, are not detailed in the present article and we invite the reader to consult them for an overview on the potential of this technology, especially in the medical field [4–21]. The general principle of the Fiber Evanescent Wave Spectroscopy is based on the fact that the light propagating in the optical fiber provides an evanescent wave at the fiber interface with air [24]. If a chemical or biological species is in direct contact with the fiber and has absorption bands in the IR spectral region, the evanescent waves will be partially absorbed at each reflection leading to a reduction of the fiber transmission which can be then measured. The black body source light of a Fourier Transform InfraRed spectrometer (FTIR-vector 22, Bruker, Billerica, MA, USA) is focused at the input of the chalcogenide glass fiber. The fiber transports the light from this source to the sample, and brings back the signal to the mercury cadmium telluride (MCT) detector of the FTIR spectrometer. Thus, a single fiber is used for the mid-infrared light transportation and as sensing transducing head.

### 2.2. Fabrication of the Initial Optical Fiber

For all the works mentioned in the Introduction, selenide glass optical fibers have been used. Among the numerous selenide glass compositions, Te_2_As_3_Se_5_ (called TAS) appeared as an interesting compromise maintaining the desirable mechanical properties associated with the glass transition temperature value T_g_ = 137 °C [1,2]. For higher temperature applications, it is possible to manipulate the glass composition [15]. Nevertheless, the TAS glass offers a large spectral window, lying from 2 to 16 µm as a bulk, and exhibits excellent resistance to crystallization. This is a key point to be able to shape it first into an optical fiber, which will furthermore be shaped again as describes in Section 3.2. Also, to obtain a perfect optical transparency, special attention has to be paid to the purification of the glass. Thus TAS glasses are synthesized under vacuum in silica vessels from single elements (Te, As and Se) of high purity. To avoid any contamination by hydrogen and oxygen impurities, the elements are repurified following a severe protocol and the mixture is distilled to eliminate carbon and silica impurities. Then, the fibered sensors, which are basic single index fibers, are simply fabricated by drawing the glass at high temperature thanks to a homemade fibering tower, especially devoted to chalcogenide glasses, which are considered soft glasses. Such TAS glass fibers transmit properly the light from 2 to 12 µm with a minimum of attenuation (0.5 dB·m^−1^) from 6 to 9 µm [25]. This value, far from that of silica fiber (0.15 dB·km^−1^ @ 1.55 µm), is sufficiently low for short range applications such as FEWS, and, overall, this fiber transmits light up to 12 µm while silica fibers are blind beyond 2 µm. Note also that the refractive index of the TAS glass is equal to 2.8, much higher than for silica, consequently, TAS glass fibers are highly multimode [25].

### 2.3. Fabrication of the Looped Sensing Head

**Figure 1.**
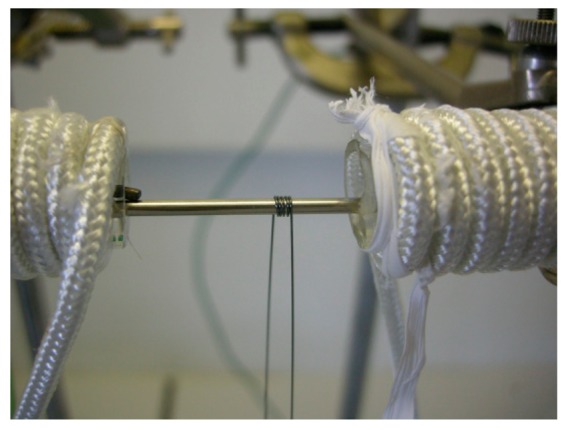
Photograph of the shaping set-up with the loop-shaped optical fiber. The steel mandrel is heated above the Tg of the glass thanks to the resistive elements on both sides.

The very good stability of the TAS glass toward crystallization offers the possibility of reheating the glass until its softening point without any optical or mechanical degradation of the fiber, so in order to shape the looped sensing head, the fiber is rolled up along a metallic stem. To achieve this goal, a special furnace has been fabricated. It is constituted by two resistive elements and a steel mandrel with appropriate diameter. Thanks to the resistive elements, the mandrel is heated until the Littleton softening point of the glass, *i.e.*, the η viscosity around 10^6.6^ Pa.s. The initial fiber with a diameter equal to 250 µm is laid down on the mandrel and, when the glass fiber reaches the ideal viscosity, the fiber is rolled up around the steel mandrel. A photograph of the set-up is shown in Figure 1. Following this procedure, one or several U-turn can be done, constituting the looped head of the fiber sensor. To conclude, the temperature is decreased until the transition temperature during a couple of minutes in order to anneal the looped fiber and then cooled down to room temperature.

### 2.4. Mechanical Tests

**Figure 2.**
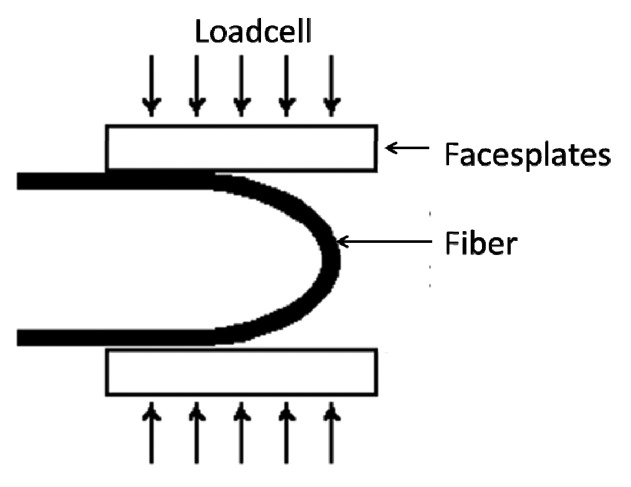
Schematic representation of the two-points bending test.

To implement the mechanical tests, a two-point bend tester has been used, see Figure 2 [26]. This test is an extremely convenient technique for determining the strength of fibers. This compressive strength testing is realized with a LR50K device (Lloyd Instrument, Elancourt, France) coupled with Lloyd Instrument 1 kN load cell. The fiber is positioned between two faceplates. The faceplates are grooved to hold the fiber in the proper position. The fiber can be either a single U-turn, or a fiber head with one or several shaped loops, as explained above.

## 3. Results and Discussion

### 3.1. Looped Sensing Head Fabrication

**Figure 3.**
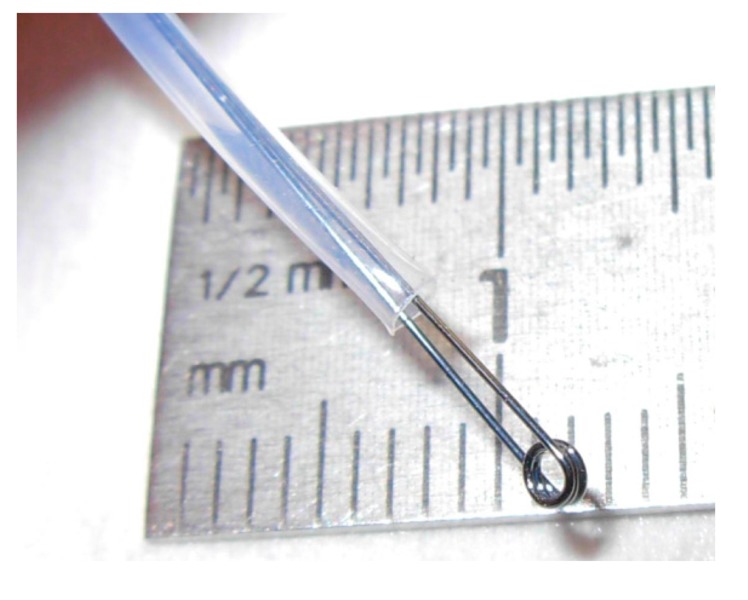
Photograph of a sensing head with one complete chalcogenide glass fiber loop.

Following the procedure described in Section 2.3, several sensing heads with one, two or three loops have been fabricated. Typically the fiber diameter is 250 µm and the external diameter of the head can be decreased to about 2 mm (see Figure 3). No degradation of the fiber surface is observed.

To check the optical abilities of the sensing head and the consequences of the shaping of the fiber, the final system has been tested using an alcoholic solution as target sample.

### 3.2. Looped Sensing Head Sensitivity

**Figure 4.**
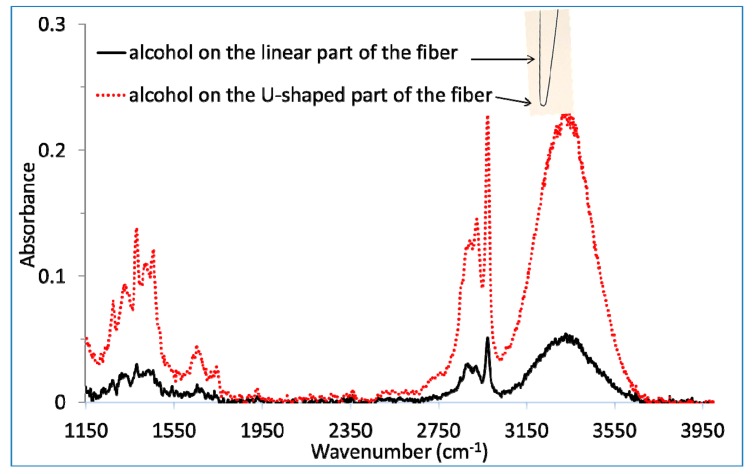
Comparison between two alcoholic solution spectra depending on the position of the drops, on the U-turn or on the linear transportation zone.

The first measurements were carried out on a single U-turn shaped fiber with a very small curvature radius equal to 1 mm (inset in Figure 4).

As a first step, a drop of an alcoholic solution, typically 10 µL, has been deposited on the linear part of the fiber as represented in Figure 4. This measurement shows that the fiber with a single U-turn presents a transmission zone between 2 to 12 µm, equivalent to the initial fiber. It allows detecting the three specific absorption bands of ethanol around 1350 cm^−1^, 2900 cm^−1^ and 3350 cm^−1^ (see Figure 4) showing that the shaping at high temperature of the fiber did not degrade the mid-infrared transparency of the fiber.

Second, an equivalent drop was carefully deposited on the U-turn keeping the same area of liquid in contact with the fiber than for the first measurement. The spectra, presented in Figure 4 show that the sensitivity is not at all affected by the propagation of the light in the tiny U-turn. This sensitivity is even about five times higher than for the drop deposited in the linear part for an equivalent surface area. Two assumptions can be proposed to explain this observation. First, the number of internal reflections for a given fiber length could increase for such a small curvature radius. Second, the penetration depth of the evanescent wave is positively affected by this curvature, which explains how the interaction of the beam becomes stronger with the sample. This assumption has been verified experimentally and theoretically by Gupta *et al.* [27]. They show that the sensitivity increases when the bending radius decreases. To better understand this observation and verify these assumptions, some simulations of the beam light propagation in such a U-turn would be useful. Anyway, at this stage, this first set of measurements is promising.

**Figure 5.**
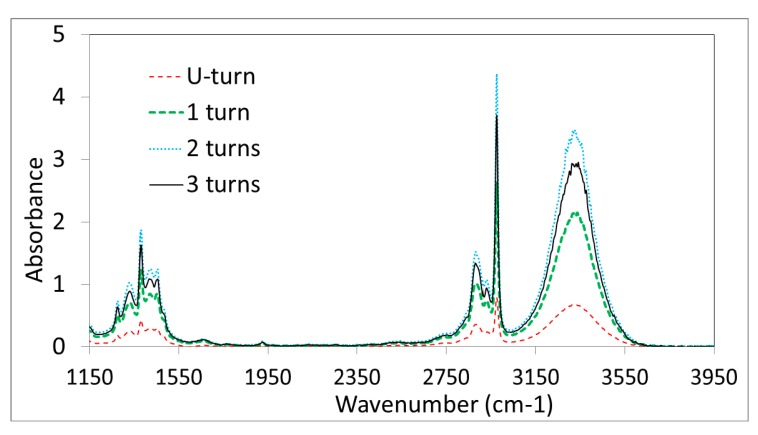
Evolution of the sensitivity (still with an alcoholic solution) with the number loops of the sensing head: a single U-turn, one, two and three full loops or turns.

The aim of the second set of measurements is to observe the evolution of the sensitivity *versus* the number of loops. Figure 5 shows the evolution of the absorbance of an equivalent drop of alcoholic solution deposited on four sensing heads with different designs: a single U-turn, one, two and three full loops of fibers with a radius curvature equal to 1 mm. For the four measurements, the drops were brought in contact with the whole looped head as for a final user of the sensor in real life conditions. Therefore, it is quite logical to observe in Figure 5 an increase of the global sensitivity with the number of loops from 0 to 2 full loops. Indeed, the contact length between the fiber and the sample also increases with the number of loops.

Interestingly, the sensitivity starts to decrease for three loops. This is probably due to the optical losses provoked by the looping-shape of the fiber. Indeed, when no sample is in contact with the fiber, the total energy of the light at the fiber output decreases with the number of loops, so a compromise has to be found between these optical losses on one hand and the efficiency of the interaction with the sample on the other hand. Clearly, two full loops correspond to this best compromise. The second motivation for shaping the sensing head is also to improve the mechanical strength of the fibered sensor.

### 3.3. Looped Sensing Head Mechanical Properties

Different designs of the sensing head have been compared using a 2-point bending test: a classical TAS glass fiber, a fiber head tapered down to 100 µm, a single U-turn head shaped as explained in Section 2.3, and a head constituted of one full loop. The results are gathered in Table 1.

The breaking tests show that tapering provides very brittle fibers with a breaking load around 0.01 N. On the other hand, the breaking load increases strongly for the head shaped at its softening temperature around the metallic mandrel. For a single U-turn, the load is 50 times as large as with the classical fiber, and even 130 times higher for one complete loop. These thermically shaped fibers become easy to handle for any final user with limited breakage risk. Furthermore, note that for the tapered fibers (which are commonly used at this time), to the breaking radius is 5 mm. In service, the radius is equal to 15 mm at best, far from the compactness of the U-turn or loop shaped sensing head described herein. If needed, a loop radius smaller than 1 mm could even be shaped.

## 4. Conclusions

Some compact sensing heads have been fabricated with chalcogenide glass optical fibers. It is possible to shape a looping head with a diameter equal to 2 mm. This achievement is really outstanding considering the known brittleness of such uncoated fibers. It has also been shown that this thermo-mechanical shaping does not degrade the sensitivity of the FEWS experiments which are even higher than with classical fibers. Moreover, the breaking loads of the loop shaped head are also much higher than with tapered fibers, which are usually used for mid-infrared FEWS experiments.

In conclusion, the realization of this compact and sensitive measuring head paves the way for the use of mid-infrared technology during medical operations, *in situ* and even *in vivo*. Indeed, we have already shown in the past the potential of this technique in microbiology and medicine, it now becomes possible to slide an optical fiber in the operating channel of a standard catheter of 2.8 mm diameter.

## Figures and Tables

**Table 1.** Results of the 2-point bend tests for four different fibers.

	**Fiber Diameter ± 5 µm**	**Bending Radius ± 0.5 mm**	**Load before the Reak**
Classical fiber	240	20 mm	0.03N ± 0.005 N
Tapered fiber	100	5 mm	0.01N ± 0.005 N
Fiber with U-turn	240	1 mm	1.5N ± 0.2 N
Fiber with 1 loop	240	1 mm	4N ± 0.2 N
